# Digital Image Analysis of Heterogeneous Tuberculosis Pulmonary Pathology in Non-Clinical Animal Models using Deep Convolutional Neural Networks

**DOI:** 10.1038/s41598-020-62960-6

**Published:** 2020-04-08

**Authors:** Bryce C. Asay, Blake Blue Edwards, Jenna Andrews, Michelle E. Ramey, Jameson D. Richard, Brendan K. Podell, Juan F. Muñoz Gutiérrez, Chad B. Frank, Forgivemore Magunda, Gregory T. Robertson, Michael Lyons, Asa Ben-Hur, Anne J. Lenaerts

**Affiliations:** 1 Mycobacteria Research Laboratories, Department of Microbiology, Immunology and Pathology, Colorado State University, Fort Collins, Colorado United States of America; 2 Department of Computer Science, Colorado State University, Fort Collins, Colorado United States of America

**Keywords:** Animal disease models, Machine learning

## Abstract

Efforts to develop effective and safe drugs for treatment of tuberculosis require preclinical evaluation in animal models. Alongside efficacy testing of novel therapies, effects on pulmonary pathology and disease progression are monitored by using histopathology images from these infected animals. To compare the severity of disease across treatment cohorts, pathologists have historically assigned a semi-quantitative histopathology score that may be subjective in terms of their training, experience, and personal bias. Manual histopathology therefore has limitations regarding reproducibility between studies and pathologists, potentially masking successful treatments. This report describes a pathologist-assistive software tool that reduces these user limitations, while providing a rapid, quantitative scoring system for digital histopathology image analysis. The software, called ‘Lesion Image Recognition and Analysis’ (LIRA), employs convolutional neural networks to classify seven different pathology features, including three different lesion types from pulmonary tissues of the C3HeB/FeJ tuberculosis mouse model. LIRA was developed to improve the efficiency of histopathology analysis for mouse tuberculosis infection models, this approach has also broader applications to other disease models and tissues. The full source code and documentation is available from https://Github.com/TB-imaging/LIRA.

## Introduction

Tuberculosis (TB) is a communicable disease which continues to affect modern society with a quarter of the world believed to have active or latent TB, primarily in low and middle income countries^1,2^. TB is transmitted by aerosol inhalation of the bacterium *Mycobacterium tuberculosis* (Mtb) from an infected individual. During the course of infection, a wide variety of pulmonary disease lesion presentations may concurrently present within the same host^3–5^. This pulmonary pathology includes, but is not limited to, inflammatory lesions, interstitial pneumonia, necrotic caseating granulomas encapsulated within a fibrotic rim, non-cavitary necrotic lesions, non-necrotic cellular lesions, and cavitary lesions^6,7^. The heterogeneity of lesions within a single individual represent a myriad of microenvironments for Mtb that range from hypoxic regions where bacteria are extracellular in caseum, to vascularized and more aerated regions where Mtb is intracellular within various macrophage populations^8,9^. Because of the complex environments that develop in the lung throughout infection, Mtb has developed adaptive strategies to alter its metabolism and replicative state in order to increase survival^10–13^. The adaptation of Mtb to its environment results in bacterial populations exhibiting multiple distinct phenotypes^8,14^. A critical component in understanding disease outcome and, importantly, treatment success is our understanding of these varying lesion microenvironments and bacterial phenotypes^15–17^. Two important clinical implications emerge from the lesion heterogeneity observed in lungs of TB patients in terms of the effectiveness of drug treatment in the various lesion types and lesion compartments. Lesion characteristics differentially affect (1) drug penetration and distribution of TB drugs inside the lung lesions where the bacteria are located^18^, and (2) the relative drug susceptibility of the bacilli contained within these lesions due to metabolic adaptation that could induce phenotypic drug tolerance and bacterial persistence^8,19^. These combined factors result in marked inter- and intra-lesional variability of drug-mediated killing. We have reported previously on the C3HeB/FeJ mouse model, which after Mtb aerosol infection, develops a heterogeneity of pulmonary lesion types more reflective of the lung pathology seen in TB patients, and which has proven useful to further our understanding of disease progression as well as studying treatment responses for emerging therapies^13,20–23^.

Murine models are employed in TB research due to their small size and low cost, and in addition, their physiology and genetics are well understood. Standard laboratory mouse strains such as C57BL/6 and Balb/c, have been used most widely for TB research. However, these mouse strains show limitations by solely developing a single lesion type after Mtb infection and they lack the lesion heterogeneity seen in TB patients. The C3HeB/FeJ TB infection model presents with a heterogeneity of pulmonary lesion types more reflective of human disease^20,21^. This mouse model was first described for tuberculosis by Igor Kramnik *et al*. (and is therefore also more commonly referred to as “the Kramnik mouse model”)^24^. These mice developed a *de novo* recessive allele, identified in a region at the 54.0-cM location of chromosome 1, termed the ‘super susceptibility to tuberculosis -1’ locus (sst1)^25^. The susceptible sst1 allele was reported to control the formation of caseous necrosis of pulmonary lesions. In C3HeB/FeJ mice after an Mtb infection with a low inoculum, the bacterial load increases to high bacterial numbers in lungs, and mice develop a chronic disease state with a dramatic progression of the lung pathology over time. After 8 weeks post infection, three pulmonary lesion types can be identified which we classified earlier^20,21^, as: (i) a highly organized encapsulated caseous necrotic granuloma defined as a Type I lesion, (ii) a neutrophil dominated lesion with cellular necrosis defined as a Type II lesion, and (iii) cellular lesions with distinct clusters of lymphocytes defined as a Type III lesion type. Both the Type I and II lesion types contain high bacterial numbers (up to 10^6-7^ per lesion), whereas the smaller Type III lesions only show few bacteria (up to 10^2-3^ per lesion). All three lesions types can be present at the same time within a single mouse, and even in a single lung lobe^21^. Occasionally cavities have been observed, although this has been an infrequent event in the C3HeB/FeJ mouse model infected with Mtb H37Rv or Erdman^20,26^.

The efficacy of tuberculosis vaccines^27,28^ or drugs^29,30^ is primarily measured by determining the reduction in bacterial load in target organs. The quantification of lung involvement in disease has proven to be informative as a secondary readout. A thorough pathology assessment can be informative to assess the effect of the therapeutic intervention on the disease itself, by studying improvement or worsening of inflammation or measures of tissue repair, such as fibrosis, as well as revealing potential immunotoxicities. Serial positron emission tomography (PET) and computed tomography (CT) scans of *M*. *tuberculosis*-infected animals and TB patients have been used to monitor disease progression and response to treatment^31–33^, allowing precise quantitation of the extent of disease and inflammatory response of the host. Parallel studies of cynomolgus macaques and humans have shown comparable rates of radiological response to linezolid^34^, and based on these initial studies, radiographic surrogate markers are being actively explored for use in human clinical trials of new agents and TB regimens^35–37^. For mouse models used in preclinical testing for TB, the treatment effect on pulmonary pathology is generally examined on microscopic images using a histological grading system by a veterinary pathologist specialized in TB^38^. The pathologist then assesses both macroscopic recognition of larger pathologic structures such as lesion types, as well as microscopic identification of predominate immune cell types. Because of the complex nature of the classification process, quantification of the lesions and their lung involvement for research purposes is not only a specialized task but highly time-consuming and variable upon the individual performing the analysis. The person-to-person variability and subject nature of the method can lead to differing results, and this will in addition make inter-experiment comparisons of the histopathology results difficult. The manual time-consuming analysis can lead to increased user fatigue and reduced focus, which in itself can lead to introduction of more inaccuracies.

While histopathology analysis of TB lung lesions is complex, recent advances in machine learning can provide for certain aspects of such analyses to be automated. Areas such as digital image analysis in cancer research^39–41^, magnetic resonance imaging/computed tomography^42,43^, and clinical and anatomical pathology^44,45^ have seen an increase of analysis tools being developed using machine learning approaches. Many of the algorithms currently employed in the medical digital imaging field include convolutional neural networks (CNN)^46–49^, and support vector machines (SVM)^50,51^. CNNs are neural networks designed specifically for images, and address the issue of achieving classification invariance for object recognition using local feature extractors or filters. These feature extractors or filters are applied at increasing levels of granularity, thereby allowing the system to recognize features at increasing levels of abstraction similarly to how the brain processes visual stimuli^52,53^. CNNs also have a demonstrated history of success in image classification, and a plethora of open source tools and packages are widely available for use^54^.

**Figure 1:**
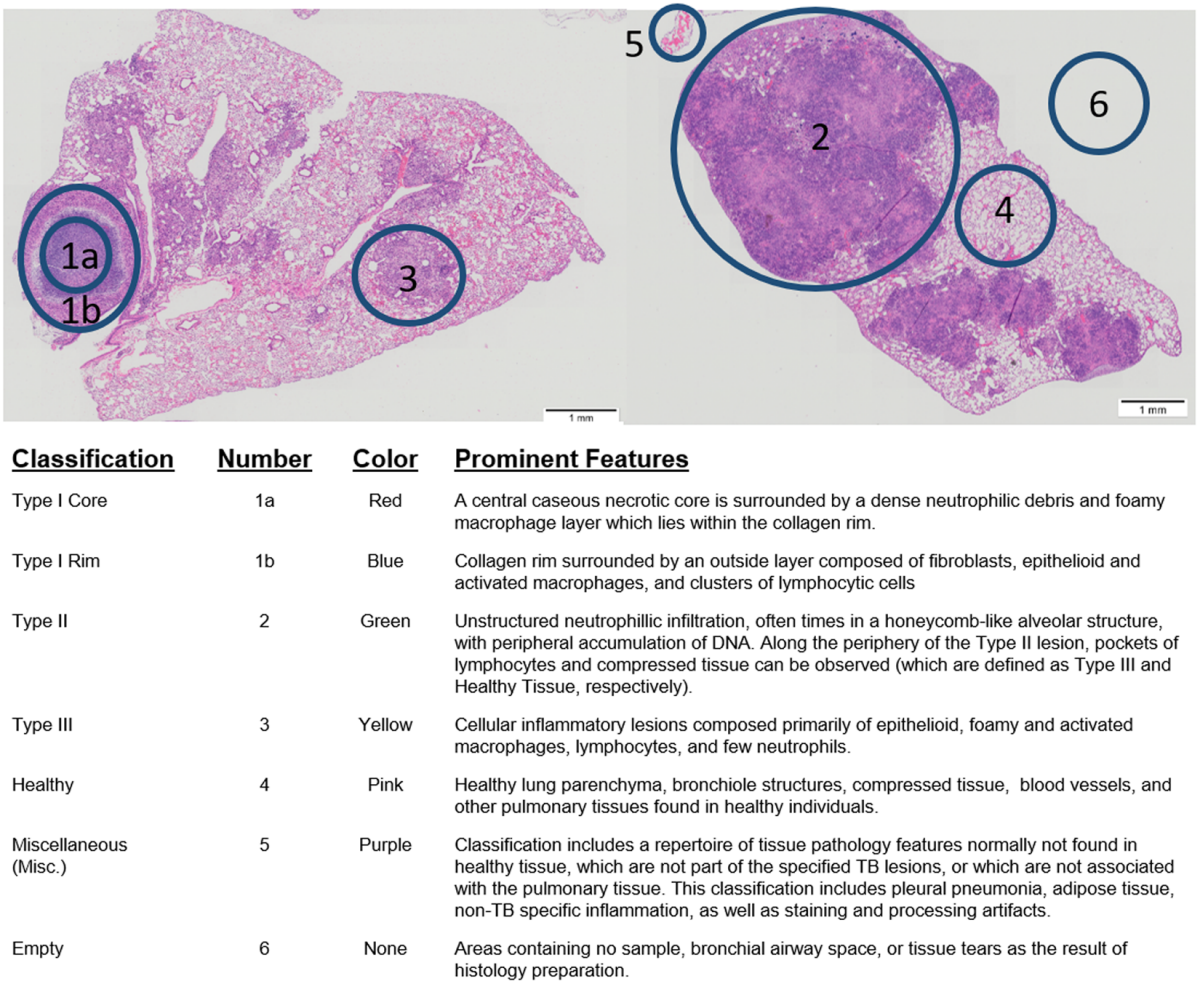
Description of the various classifications in C3HeB/FeJ lung pathology used in LIRA.

**Figure 2:**
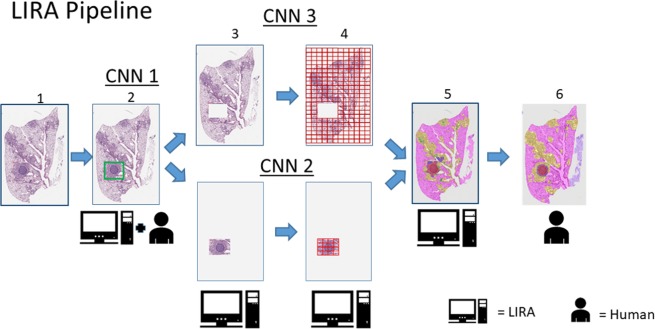
Graphical representation of the LIRA digital imaging analysis workflow. (1) The original digital image scan is uploaded, (2) CNN1 detects Type I pulmonary lesions and results are verified by the user, (3) Window location is cropped based upon classification, (4) Image is tiled in individual image patches of 80 × 145 pixel size to generate predictions, (5) Classification predictions generated using both CNNs, (6) User is allowed to edit the results one last time.

In this work, we propose a TB lesion machine learning classifier that follows established classification criteria developed in our laboratory which distinguishes three different lesion types that develop in the C3HeB/FeJ mouse TB infection model^20,21^. This digital image analysis software package was developed as a modular neural network^55–58^, consisting of three CNNs, each optimized for a specific sub-task, together with two human intervention checkpoints to limit the probability of misclassification (Figs. 1, 2). This digital image analysis pipeline is named ‘Lesion Image Recognition and Analysis’, or LIRA. From its inception, LIRA was designed to work in conjunction with pathologists with the goal to improve the analysis of pulmonary pathology to be more efficient, accurate, and reproducible regardless of the individual analyzing the whole slide images.

## Results

### Ability of CNN1 to identify caseous necrotic (Type I) lesions

After training CNN1 with the digital image training set, a validation step was introduced to evaluate the sensitivity, specificity and false positive rate of classifying Type I lesion cores. After an image is uploaded, the Type I object detector (CNN1) is used to macroscopically detect the caseous necrotic center of Type I lesions (Figs. 1,2). LIRAs CNN1, the Type I object detector, showed to be consistent in identifying and delineating Type I lesion structures, with a sensitivity of 86.36%. CNN1 however shows a high false positive rate by misclassifying other lesion types for a Type I with the specificity of 55.11%. Most misclassifications occurred when large areas of cellular necrosis were visible in the center of Type II lesions, which on a single image patch shows a similar cellular composition as the Type I cores. The high recall rate is likely the result of the training set for the creation of CNN1, having an insufficient number of images needed for training. To decrease the false positive rate for CNN1, continued training with a larger training set might be an area of improvement for future iterations. Despite the high recall rate, we opted to retain CNN1 as it facilitated the rapid identification of Type I lesions for the user, and it provided an accurate demarcation of its margins from surrounding lesions or tissue. For instance, the validation results showed that the inclusion of CNN1 decreased the inconsistencies seen after hand labeling among multiple pathologists in identifying and demarcation of the Type I Rim. However, it is possible that repeated false positive results could sway the less experienced user to misclassify over time. Therefore extra scrutiny is needed during the CNN1 step to limit potential bias of less experienced users which could lead to an increase of the number of false positives. Overall, the addition of CNN1 proved to be a substantial time saver as more time is allocated to accurately identify the margins of the Type I lesions than in the removal of a potential misclassification.

### The accuracy for LIRA in comparison to an experienced TB researcher

After the initial positive or negative classification by the macroscopic object detector CNN1, the areas designated as Type I were further classified by CNN2, and the remaining image patches were classified in parallel by CNN3 (Figs. 1,2). To determine the accuracy of the overall pipeline (Fig. 2), a ground truth was established on 12 digital images by a researcher with substantial experience analyzing C3HeB/FeJ mouse pathology. These 12 images consisted of lung lobes composed of three Type I dominated tissues, three Type II dominated tissues, three Type III dominated tissues, and three predominately Healthy tissues. Total image patch counts for only the classification of interest were used to calculate the percent error per individual classification. The results for LIRA without human intervention were then compared to the manual labeling results by the researcher. As seen in Table 1, the overall percent error for all seven classifications was 12.11% across all 12 digital images. Healthy Tissue and Type III had the lowest percent error, while Type II had the highest percent error at 38.37%.

**Table 1:** Accuracy of the LIRA predictions for the various lesion classifications measured by percent error agreement. N: number of digital images analyzed.

	LIRA % Error
Healthy Tissue (n = 3)	1.975
Type I - Caseum (n = 3)	17.324
Type I - Rim (n = 3)	8.324
Type II (n = 3)	38.374
Type III (n = 3)	2.829
Total (n = 12)	12.107

The high percent error observed for the Type II lesions for LIRA compared to the experienced researcher was the result of poor demarcation by LIRA of the surrounding lesion tissue, which is often composed of compressed tissue and in some cases aggregates of extracellular DNA (potentially NETs). Compressed tissue results from lesions pressing into healthy lung parenchyma, which changes the morphological structure. The aggregates of extracellular DNA are considered part of a Type II lesion, whereas compressed tissue as defined here is considered “Healthy Tissue”. Another area contributing to the decrease in accuracy by LIRA was from processing artifacts to generate the microscope slides. These included areas of high red blood cell numbers, which were incorrectly classified by LIRA as Type III. Also, minor differences in the staining procedure of the microscope slides affected the intensity and colors of the tissue and its images, which resulted in slight differences in classification by LIRA. Lastly, sectioning artifacts such as microtome occasional knife chatter, lines, or fragment holes were also prone to result in misclassification by LIRA.

### Improved agreement of histopathology classifications among pathologists using LIRA

**Figure 3:**
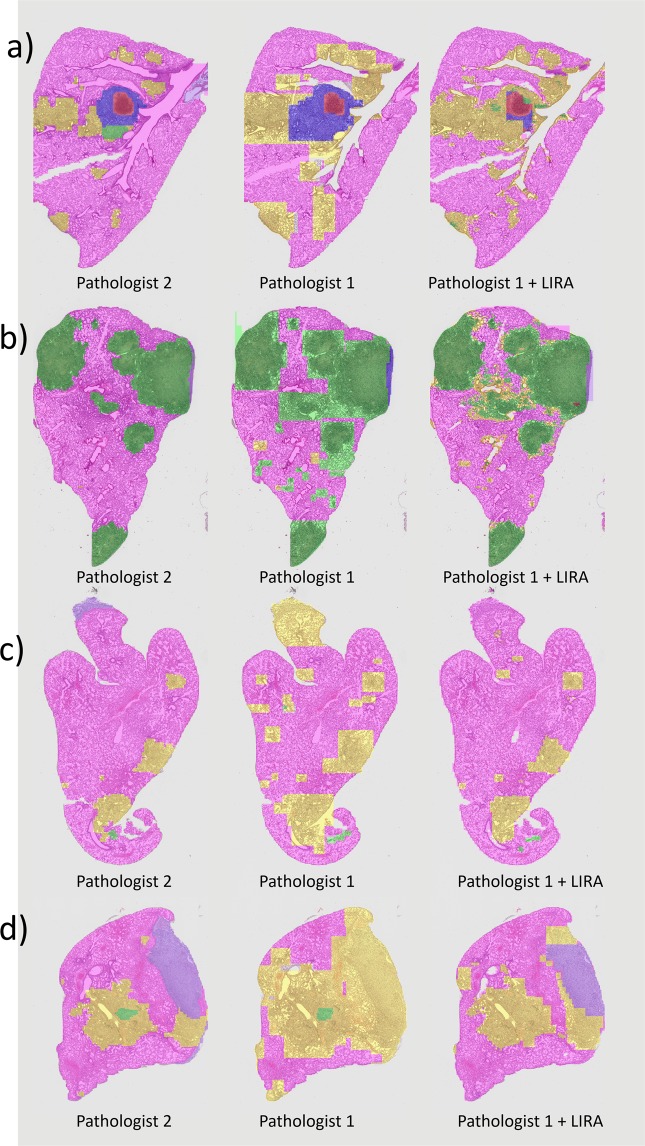
Digital Image Histopathology Data from Pathologists With and Without LIRA assistance. Color overlay results of 4 images from the image validation set with **(a**) Image 3, (**b**) Image 5, **(c**) Image 2, and (**d**) Image 7. The far left images are from pathologist 2 with the most accurate hand labeled results, the middle images are from pathologist 1 with the least accurately labeled results, and the images on the right hand side show the results of pathologist 1 with the assistance of LIRA.

To evaluate the performance of LIRA in terms of reproducibility and concordance between users, a second validation step was included using a previously unseen digital image set. Seven randomly selected scanned images of single lung lobes were used for this validation step. The goal for this validation step was to compare the pathology classification results from the classical pathology analysis approach of four pathologists or four research technicians, to the prediction results of LIRA with pathologist assistance (see Supplemental Fig. 1). The comparison focused on three criteria: (1) the accuracy of both approaches in identifying the correct pathology classification; (2) the accuracy of demarcation of lesion margins that affects the area of lung involvement, and (3) the variability between users. For this purpose, the pathologists were first given seven images to classify each image patch by hand. After one week, the pathologists used the same seven digital images in a random order to classify each image patch now with the assistance of LIRA. Using the classical pathology approach, results of the four pathologists showed a consensus on the specific lesion classification in general, but did not always agree on the extent of the percent lung involvement (Supplemental Fig. 2). The disease classification categories with the most variability in results were Type I Rim and Type II lesion types, which subsequently also impacted indirectly the Healthy tissue classification. The variation in results within these categories was mostly the result of inaccurate demarcation of lesion margins after manual labeling by the pathologists. Specific examples detailing the high variability for each of these classifications are presented in Fig. 3 and Supplemental Figs. 1 & 2.

As LIRA makes classification predictions per image patch at a microscopic level, it became clear in the validation step that both CNN2 and CNN3 can identify certain areas of a single lesion as one classification whereas another area of the same lesion could be identified as a different classification. An example of this can be observed in Supplemental Fig. 2 (Image 3), which consists of one Type I and multiple Type III lesions. In this example for the pathologist-only readout, an observed disagreement was seen for the demarcation of the Type I Rim and the start of the Type III lesion that is adjacent to it. Only one of the four pathologists (pathologist 2) had accurately identified neutrophil-rich regions of the Type III lesion (in green), which is an unusual event for a Type III lesion. For the pathologist-assisted LIRA results (Supplemental Fig. 2), three out of four pathologists decided to reduce the lung area for the Type I lesion after being prompted by LIRA. In addition, all four pathologists recognized regions of neutrophil infiltration only after the assistance of LIRA (in green). Another disagreement between pathologists was observed regarding the classification of bronchial airways and artifacts. For the pathologist-only readout, two out of four pathologists classified the bronchial airways as Healthy tissue, and the two remaining pathologists classified these regions as Empty slide. The same was observed for a tissue processing artifact visible on the image scan (a fragment hole artifact), which was classified by two pathologists as Healthy tissue and by the other two as Empty slide. With the assistance of LIRA, the results for all four pathologists were now in agreement, and both the bronchial airways and the artifact were classified correctly as Empty slide. The change in classification that occurred at the pathologists’ checkpoints after CNN2 and CNN3 showed to have a significant impact on the output data in terms of reducing the variability and increasing reproducibility of the results.

Reproducibility of histopathology analysis regardless of the pathologist analyzing the data, or the variation that can occur over time is important when comparing data across preclinical studies or laboratories. To measure whether the agreement between pathologists increased with the assistance of LIRA, multi-rater percent agreement and the Nominal Krippendorff’s alpha approach were used. Each image patch was considered a separate observation for the analysis, and the percent agreement was calculated per image. The multi-rater average percent agreement without LIRA for the four pathologists across all images was 87%. Images showing the lowest agreement consisted of primarily Type I (Images 1&3) and the Misc. classification (Image 7) (Table 2 and Fig. 3D). With the assistance of LIRA, the percent agreement for the four pathologists increased across all images, and there was a 53% decrease in disagreement overall to achieve an overall agreement of 94%. The most prominent increase in percent agreement was observed for the images containing Type I lesions and the Misc. classification.

**Table 2:** Agreement of the pathologist readouts with LIRA assistance (Path+LIRA) and without (Path) by measuring percent agreement and Krippendorfs alpha.

Image	Percent Agreement		Krippendorfs Alpha	
	*Path*	*Path + LIRA*	*Path*	*Path + LIRA*
1	84%	94%	0.761	0.904
2	92%	97%	0.864	0.951
3	84%	94%	0.757	0.902
4	92%	94%	0.860	0.903
5	90%	95%	0.849	0.920
6	84%	93%	0.757	0.891
7	82%	93%	0.725	0.896
Mean	87%	94%	0.796	0.910

Using a more refined additional metric of analysis, the Krippendorff’s statistical analysis approach was used for calculating the nominal alpha coefficient. Again, each image patch classification by the four pathologists was seen as a separate observation. Manual labeling of seven pathology features for seven images by the four pathologists showed an average alpha of 0.796 (Table 2), which is interpreted as tentative. Four of the seven classified images were below the 0.80 threshold, which means the pathologists failed to achieve good agreement on the classification of those images. In contrast, when performing the Krippendorff’s analysis on the data generated by the four pathologists with the assistance of LIRA, the calculated average alpha was 0.91 (Table 2). The Krippendorff’s analysis results for all seven images were above the 0.80 threshold, and results for all seven images were above the 0.8 threshold which is considered good. These results confirmed our observations using percent error, providing further evidence of the benefits of LIRA assistance.

**Figure 4:**
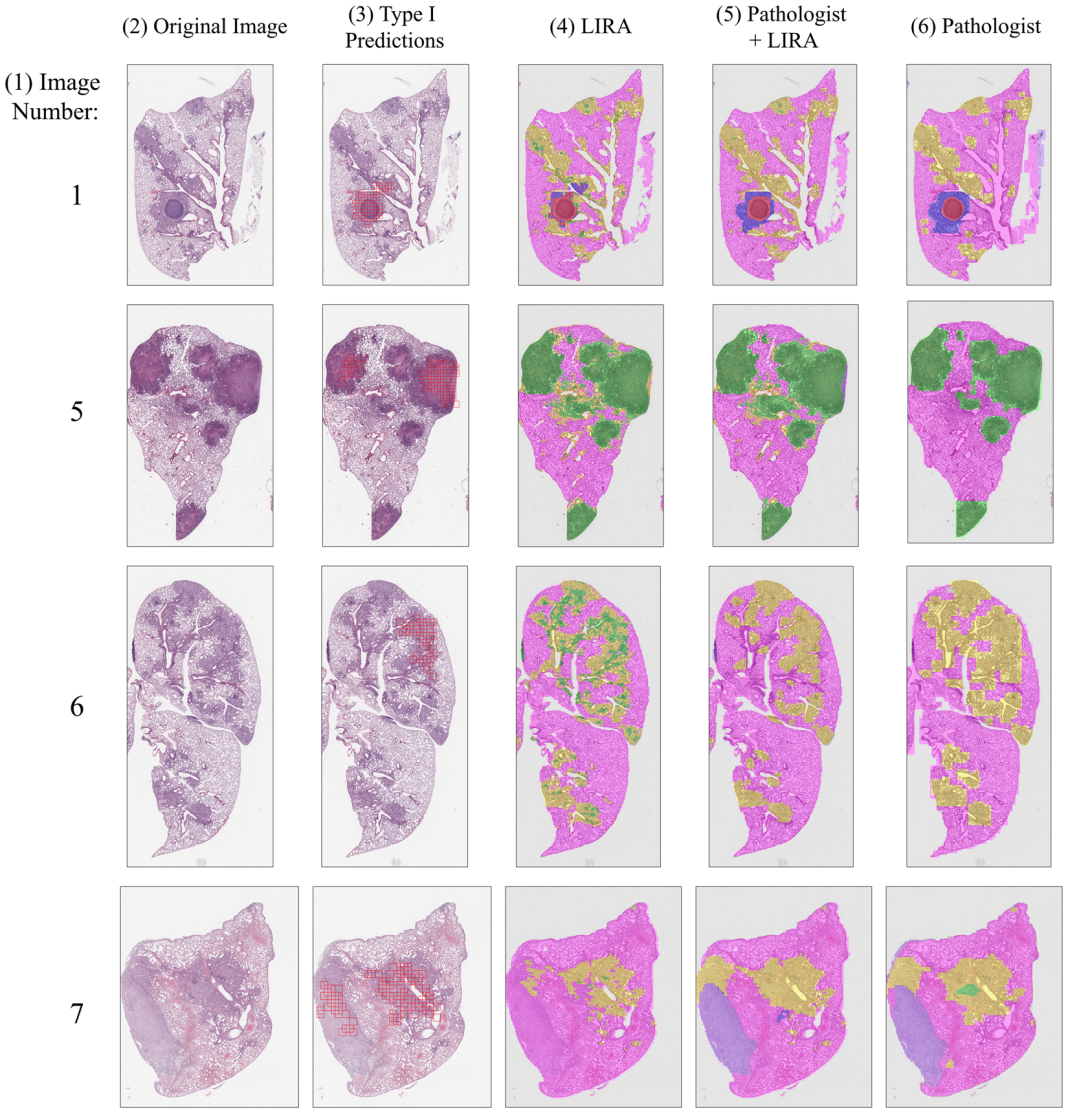
Visual representations of the classifications made during each step in LIRA using colored overlays. (1) Image number associated with the analysis, (2) the original H&E stained image without predictions, (3) The Type I object detector predictions made by CNN1 represented by red grid lines, (4) All predictions by the microscopic classifiers CNN2, CNN3, (5) Final classification changes made by user (Pathologist + LIRA), (6) Classifications made by user using manual labeling approach without software assistance (Pathologist).

The increase in agreement among pathologists with the assistance of LIRA was the result of two main beneficial factors. First, the use of the LIRA software increased the accuracy of the demarcation of lesion margins, which affected indirectly the percent lung involvement in various categories. In addition, LIRA prompted the pathologist to revisit and re-analyze specific areas in the lungs, often small in size, which were often overlooked using the classical histopathology approach. Examples are shown in Fig. 3 (Pathologist 1, Pathologist 1 + LIRA) and Fig. 4 (Images 6 & 7), which show the improvement of lesion margin demarcation with LIRA assistance. With the use of LIRA, the classification of the pathological features not only improved the agreement between pathologists, but the results tended to be closer to the predictions made by the most experienced TB pathologist (Pathologist 4) (Supplemental Fig. 1). An example where LIRA prompted the user to re-analyze their initial prediction is shown in Fig. 3D. Here, a large area of fibrotic tissue is observed, which should have been classified as miscellaneous as it does not fit any other definition. With the assistance of LIRA, the pathologist changed the classification to miscellaneous, which was in agreement with the other three pathologists. The accuracy of classifying image features in the miscellaneous category is mostly dependent on the background and level of expertise of the user, and therefore this category will always be the most difficult to fully standardize across multiple users. The assistance of LIRA in the miscellaneous category, however, did minimize the misclassifications in this category and was an added benefit.

In summary, the results of the validation tests showed a clear benefit of using LIRA assistance in the pathology readings by board certified pathologists, and this by improving the accuracy in demarcation of lesion margins as well as prompting the user to re-analyze certain areas thereby increasing conformity and reproducibility of an otherwise subjective readout. Additionally, compared to previous manual pathology analysis methods employed by the four pathologists taking about 15-30 min manual effort per lung lobe, an average decrease in manual analysis time of 82% was observed, now reducing the histopathology manual analysis to 3-10 min automated effort per lung lobe (with 5 minutes on average per lung lobe).

### Trained research technicians + LIRA

In a final validation assessment, we investigated whether LIRA could be easily employed by research technicians, and how their prediction results would compare to those made by specialized pathologists. For this purpose, four research technicians were first trained in the identification of lesion types of the C3HeB/FeJ animal model (Trained Research Technician + LIRA). The technicians were then asked to analyze the same seven digital images of single lung lobe, as those evaluated by the pathologists for the validation step. The results showed that for the research technicians with assistance of LIRA, similar classification predictions were obtained as described earlier for the pathologists. Most notable was again the accurate demarcation of the lesions with LIRA assistance (Supplemental Fig. 2). The results by research technicians assisted by LIRA exhibited a slightly higher variability, when compared to results of the pathologists assisted by LIRA. One area where the research technicians using LIRA showed less accurate results was in the identification of the Miscellaneous Tissue category. For example, in Image 7, a large area of fibrotic inflammation was not identified by the research technicians, whereas this was correctly classified by the pathologists using LIRA (Supplemental Fig. 2, Misc., Image 7). Two out of 4 research technicians correctly identified fibrotic inflammation (Supplemental Fig. 2, Image 5), which was also observed by the pathologists.

In summary, the output of the research technicians with LIRA was remarkably similar to that of the experienced pathologists using LIRA, except when uncommon or infrequent tissue types were present. Taken together, the data of the validation study indicates that even with implementation of AI, a certain level of pathology expertise is still preferred to accurately quantify certain unique lesion pathology.

## Discussion

Traditional histopathological analysis generally involves an experienced board-certified pathologist often using a semi-quantitative scoring system to identify and quantify unique disease-specific pathology features^59,60^. Specific scoring criteria are developed which can distinguish between various disease states thereby informing about disease progression or on effects of treatment intervention. These scoring criteria are then applied in double-blind histopathology analysis to reduce bias and variability. However, issues such as reproducibility between studies and variability in readouts by different pathologists still exist, and this continues to be an area of active research^61,62^. In non-clinical TB animal studies used in drug and vaccine development, studies are often long term, require multiple time points, and generally occur in more than one laboratory to confirm that novel treatments or interventions are reproducibly efficacious. To evaluate the progression of pulmonary lesions in TB animal models, a histological grading system was developed years ago, based on grading granulomatous lesions for inflammatory cell numbers and their infiltrative distribution pattern^38^. The pathologist would then calculate a mean total lesion score for individual tissues; as normal (score of 1), mild (score: 1–3), moderate (score: 3–6), severe (score: 6–8) to reflect the disease state per animal. The variability in histopathology analysis between studies, time points, and individual pathologists, owing to the subjective nature of this method, was often substantial thereby making it difficult to interpret results. In addition, studies would be time-consuming and results over time affected by user fatigue. In this report, we describe the development of novel software for a rapid automated and unbiased digital image analysis using a machine learning approach based on convolutional neural networks to evaluate the histopathology of mouse models for tuberculosis in a quantitative manner. The goal was to make the software intuitive for its user, as well as facilitate and accelerate histopathology analysis for the non-expert.

We report here on the training and validation of a model for the accurate classification of seven pathology features using histopathology images. The model uses human classifications to learn robust features from a large number of H&E stained image patches. The model is currently suitable for quantitative lesion identification of the C3HeB/FeJ mouse model for tuberculosis. Initial attempts tried to implement a single convolutional neural network to identify all seven histopathology classifications. However, significant misclassification was observed caused by observing similar cell compositions in more than one lesion type. Therefore, LIRA was created using three individual neural networks to create a single modular network in order to generate more accurate predictions, based on both macroscopic- and microscopic histopathology events. Currently, the accuracy of the model is not sufficient to rely solely on the implementation of the LIRA software without any human intervention. Two human intervention checkpoints for the user are still required: first, to confirm and potentially modify the Type I lesion prediction (CNN1) and second, to confirm the individual image patch classifications made by LIRA (CNN2, CNN3). Moreover, human intervention can help prevent the misclassification of infrequent pathology events and artifacts on which the specific CNN had not previously been trained. Noteworthy, certain misclassifications by LIRA may ultimately be beneficial to the process as they can bring a particular pathology feature to the attention of the user, and can prompt the researcher for closer analysis. Future iterations will focus to improve the model by including additional datasets for training when more studies become available to increase LIRA’s performance under all circumstances.

There were multiple advantages to using assistive software in identifying and quantifying disease states with digital image analysis. First, we observed a significant reduction in analysis time and user-fatigue whereby an 82% decrease in time was achieved for quantitative analysis in comparison to the standard histopathology scoring methodology. Second, with the assistance of the software, the variability of the predicted classifications among different pathologists was greatly reduced. Of importance, with the assistance of LIRA, a more accurate pathology comparison will be possible across multiple animal studies, different longitudinally time points, and studies performed at multiple laboratories. Where LIRA outperforms the individual pathologist is the consistent and accurate demarcation of lesion margins, as well as the detection of small regions on a microscopic level, which both substantially affect the quantitative analysis of the lesion area involvement. Pathologists and LIRA in most instances both correctly classify the lesion type, but the variability in the results from classical pathologist readouts is derived from the imprecise or inconsistent demarcation of lesion margins. Where pathologist expertise and intervention is still needed and preferred, is for the identification of infrequent pathology events or unusual artifacts. As also described for other applications in medical image analysis, diagnostic confidence never reaches 100% and combining machine learning and physician or pathologist experience reliably enhances system performance^63^.

During the development of the LIRA software it became apparent that the quality of tissue sample preparation can impact the accuracy of the results of the software. LIRA is affected by features such as the presence of high red blood cell numbers and processing artifacts, which led to the software incorrectly classifying these regions as pulmonary lesions instead of healthy tissue. Deviation from the sample preparation protocol presented in our methods section, including using frozen sections or changes in section thickness, will also result in a decrease in accuracy. When sample preparation varies, it is recommended that a new model be trained on image patches generated with these new protocols. In addition, the current iteration of LIRA is designed to identify lesion types for C3HeB/FeJ pulmonary tissue collected between 6-10 weeks post infection. We have tested LIRA on more commonly used BALB/c or C57BL/6 pulmonary tissues infected with Mtb, and results showed these mouse samples are also suitable for LIRA use. For the more conventional mouse models we now also opted to generate a new, simpler system. The recently developed software is similar to LIRA, but is solely composed of a single neural network that can generate classifications for TB lesion, Healthy, Unknown, and Empty, because the more traditional mouse models generally only develop one lesion type after infection with TB. Because the model is simpler, the readout will be faster than LIRA and have greater accuracy since it was designed specifically for those models. We also intend to make this code available in our open source repository. Lastly, we used multiple different image capture devices for generating the training set, however, all with similar image resolution. Although not observed in our setting, it is possible that different image variables such the resolution of the camera, image file format, and variability in staining intensities could alter the final output for LIRA. The optimal settings for LIRA at this time are a camera resolution between 0.33 to 0.5 microns/pixel with images saved as a Portable Network Graphics (PNG), image acquisition using the 20x objective, and following the staining procedure for H&E as described here.

A future direction to improve the current LIRA pipeline is to generate a new classification for the surrounding Type II tissue, and to increase the sensitivity of CNN1. LIRA predictions for Type II lesions could be significantly improved by creating a separate class for the surrounding tissue, primarily composed of aggregates of extracellular DNA and compressed tissue. And in addition, an increase of the Type I detector (CNN1) sensitivity might allow omission of the first human intervention checkpoint. The current false positive rate for the Type I core is the result of the limited sample numbers that were available at the time of training CNN1. Both an increase in the data available for training and the utilization of generative adversarial networks (GANs)^64^ and pre-trained neural networks^65^ represent possible future solutions. We are currently exploring additional machine learning and more traditional computer vision techniques to increase the specificity of the CNN1 model. This includes using pre-trained neural networks, histogram of oriented gradients (HOG), and Image segmentation. LIRA 2.0 is currently under development and will be made open source at the time after these improvements have been made.

A potential adaptation of LIRA’s architecture for use in TB preclinical models could be an enhancement beyond just classification and quantification of infection in tissues. Researchers can combine lesion classification features with additional quantifiable measurements. Of particular interest in TB drug and vaccine development is to include the mycobacterial quantification metrics of each individual lesion. The goal would be to integrate precise measurements on bacterial numbers after fluorescent staining^21,66^, as well as bacterial aggregation sizes, level of fluorescence, and the average number of bacteria as a metric of area. With the availability of matrix assisted laser desorption ionization (MALDI) imaging to assess drug levels across pulmonary lesions^67,68^, one can envision the development of a model that would integrate data sets from digital lesion pathology with bacterial metrics as well as drug exposure.

In conclusion, LIRA does not replace the histopathology analysis by pathologists but instead intends to improve the accuracy, speed, and reproducibility of the analysis. While there are certain limitations with the current model, these can easily be corrected and adjusted. The ability to more quickly and more accurately assess the effect of treatment interventions on histopathology of target organs will improve the evaluation of the efficacy of novel antibiotics and vaccines. Our demonstrated approach is not limited to TB and can be modified to include additional diseases and animal models, by creating new training sets and modifying the original architecture of LIRA to meet the needs of the researcher.

## Methods

### Sample collection from archived animal studies

The digital images from pulmonary samples used for the development of the image analysis software package were derived from archived C3HeB/FeJ mouse studies conducted for the development of the mouse model^20,21^, for drug efficacy trials using existing and novel drugs for tuberculosis^13,22^, and studies focusing on virulence of various Mtb strains (unpublished results). Lung samples were collected from 14 previous independent mouse studies, conducted over several years (from 2012-2018), and samples were obtained from two different research laboratories at CSU. Mtb strains used for the mouse infection studies included *M*. *tuberculosis* Erdman (TMC 107, purchased from ATCC), HN878 (Clinical Isolate W210, CSU, Fort Collins, CO, available at BEI resources), or H37Rv (Trudeau Institute, Saranac Lake, New York). Bacteria for mouse infections were initially grown as a pellicle and further propagated in Proskauer-Beck medium containing 0.05% Tween 80 (Sigma Chemical Co., St. Louis, MO), never extending past mid-log phase^69^. Female C3HeB/FeJ mice, 6-8 weeks of age (Jackson Laboratories, Bar Harbor, ME) were housed in a bio-safety level III animal facility and exposed to an Mtb aerosol infection using a Glas-Col inhalation exposure system^70^. Mice were euthanized by CO_2_ inhalation, between 4-10 weeks post aerosol exposure. Whole lungs were fixed by inflation with 4% paraformaldehyde (Electron Microscopy Sciences, Hatfield, PA) in phosphate buffered saline solution (PBS) via cardiac perfusion before subsequently being transferred to a 70% ethanol solution 48 hours later.

### Processing of samples to generate the digital image dataset

Fixed lung samples were further processed at either Premier Laboratory (Boulder, CO), the Experimental Pathology Facility at CSU (Fort Collins, CO), or at the Microbiology, Immunology and Pathology (MIP) Department. Pulmonary tissue samples were placed in a single cassette per mouse, then paraffin embedded and sectioned. Tissue sections (5 µm) with the largest surface area were used for further histopathology analysis and mounted on glass slides. Paraffin sections processed for Haemotoxylin and Eosin (H&E) staining underwent a Xylene bath for 10 min before undergoing gradient hydration. Samples were stained using a Leica ST5020 instrument with an initial staining step using Hematoxylin 560 (Leica Selectech, Buffalo Grove, IL) for 5 min and rinsed in tap water. Next, the slides were washed using a Define wash step (Leica Selectech, Buffalo Grove, IL) for 10 sec before being placed in a Blue Buffer Solution (Leica Selectech, Buffalo Grove, IL) for 1 min. The slides were then rinsed with Eosin for 1 min before being transferred to 96 Eosin Y 515 (Leica Selectech, Buffalo Grove, IL) for 3 min. The final step included a washing step with 96% EtOH for 6 min before being mounted using a LEICA CV5030 Mounting instrument (Leica Selectech, Buffalo Grove, IL), thereby using the Coverseal-X Xylene Mounting Media (Cancer Diagnostic Inc., Durham, NC). Slides were generated and stained at three facilities using similar, however not the exact same, protocols over a time span of several years (2012-2018). Digital image scans from microscopic slides containing C3HeB/FeJ lungs were generated from one of three image capture devices. Image scans from Premier Laboratories (Longmont, CO) were generated on an Aperio Scanscope XT digital slide scanner (Nikon, Melville, NY) at 20x magnification. At the Experimental Pathology Core (CSU), the images were acquired using an Olympus VS120 digital slide scanner (Olympus, Center Valley, PA) using the Olympus VS-ASW software (v.2.9; Olympus, Center Valley, PA) at 20x magnification. Images acquired in the MIP Department were generated on a Nikon Eclipse Ti robotic inverted microscope with a DS-Qi1Mc digital camera (Nikon, Melville, NY) at 20x magnification, using the Nikon NIS Elements AR software (v. 4.51.00; Nikon, Melville, NY). In total, 86 slides were scanned on the Aperio Scanscope, 84 slides were scanned on the Olympus VS120 and 6 images were generated on the Nikon inverted microscope. All three imaging devices used for generating whole slide images show similar image resolutions, with 0.5 microns/pixel for the Aperio XT, and <0.33 micron/pixel for the Olympus VS120, and 0.322 micron/pixel for the Nikon camera at 20x magnification. Therefore, limited variability in digitizing the results was expected by the different image capture devices used. The variability between images was mainly caused by the nature of collecting biological data and differences in sample processing (processing by different technicians, using slight modifications of H&E staining protocols). This was merely seen as an asset for developing the software since it provided a realistic level of noise or variability to our data reflecting results expected in future. In total, for the training and subsequent validation of LIRA, lung sections from 176 mice were scanned digitally to generate 176 digital image scans (containing all five lung lobes for every mouse per microscopic slide and per digital image scan). To reduce the file size of the image scans, digital image files were generated for every individual lung lobe (resulting in five digital image files per mouse).

### Lesion and histopathology classification scheme

The histopathology classifications used for the creation of the training, test, and validation sets included the original classifications of pulmonary lesion types in the C3HeB/FeJ mouse model, as previously described by Lenaerts *et al*.^20,21^. Briefly, C3HeB/FeJ mice present with three distinct pulmonary lesion types after an Mtb aerosol infection. Type I lesions show well organized, caseous, necrotic lesions with a layer of foamy macrophages around a core composed of neutrophilic debris, which is surrounded by a collagen rim with interstitial macrophages admixed within the rim. Type I lesions contain high bacterial numbers, which are either extracellularly located in the lesion core, or intracellular in foamy macrophages. The Type II lesions are less organized with a massive recruitment of neutrophils, resulting in large areas of alveolar wall necrosis throughout the lung parenchyma. Type II lesions also contain high bacterial numbers, both intra- and extracellularly. Type III lesions develop as a result of accumulation of lymphocytes, epithelioid, and foamy macrophages as well as small pockets of neutrophils. The latter is similar to the main lesion type described for other immunocompetent mouse strains such as the BALB/c and C57BL/6^71–73^.

In addition to the lesion classifications, other histopathology categories were added for the machine learning approach in order to ensure inclusion of healthy tissue, non-Mtb specific lung pathology, artifacts, and an empty slide feature. In total, seven classification categories were identified, which were as follows: Type I Rim, Type I Core, Type II, Type III, Healthy, Miscellaneous (Misc.), and Empty slide. Each classification is characterized by certain macroscopic pathology features, as well as microscopic features based on multiple different cell types. Each of the seven classifications are described in detail below, and are presented in Fig. 1.

The Type I (caseous necrotic) pulmonary lesion type is characterized by two distinct pathology features which include a collagen rim and a caseous necrotic core. For this reason, the Type I lesion was categorized into two distinct classifications for the analysis, defined here as the ‘Type I Rim’ and ‘Type I Core’ categories. The Type I Core is composed of central necrosis surrounded by a dense neutrophilic debris layer, and a foamy macrophage layer which lies within the collagen rim. The Type I Rim composition includes the collagen rim and the outside layer composed of fibroblasts, epithelioid, and activated macrophages, and clusters of lymphocytic cells. The ‘Type II’ pulmonary lesion consists primarily of large numbers of neutrophils at times visible as a honeycomb-like structure of lung parenchyma. Along the periphery of the Type II lesion, the presence of aggregates of extracellular DNA, pockets of lymphocytes, and compressed tissue can be present. These pathology features are mostly found in close proximity and therefore also classified as Type II. The ‘Type III’ pulmonary lesions are cellular inflammatory lesions composed primarily of epithelioid, foamy and activated macrophages, large numbers of lymphocytes, and small pockets of neutrophils. The ‘Healthy’ classification is here reserved for tissue features, such as healthy lung parenchyma, bronchiole epithelium, blood vessels, compressed tissue, and any other pulmonary tissues that are present in healthy individuals. The ‘miscellaneous’ classification encompasses a broad array of tissue pathology features that are not found in healthy tissue, that are not part of the specified TB lesions, and are not associated with healthy pulmonary tissue. The miscellaneous classification includes pleural pneumonia, adipose tissue, non-TB specific inflammation, as well as staining and processing artifacts. Lastly, the ‘empty slide’ classification was included as a separate category, although this classification was ultimately not included in the final performance and accuracy analysis of the results.

### The LIRA pipeline

LIRA was developed as a modular neural network. This implementation of multiple neural networks working together follows the natural logic employed by trained researchers to classify lesions by intuitively first investigating on a more general, macroscopic level (for Type I lesion recognition), followed by a more detailed analysis on a microscopic level to identify other pathological features on a cellular level. In preliminary work, we used a single CNN to identify all seven classifications. However, due to similarities between multiple lesion types at a microscopic scale (such as the necrosis encountered in a Type I core and a Type II lesion), multiple neural networks operating at different visual scales (macro and micro) were used to limit misclassification of lesion types. No preprocessing of the images was employed in either training of the models or within the pipeline. The final approach, illustrated in Fig. 2, employs a modular architecture^55–58^ with three CNNs and two human intervention checkpoints. After a digital image is uploaded, in a first step the macro-classifier (CNN1) identifies the Type I physiological structure with its characteristic caseous necrotic center and collagen rim. Human feedback is required after this initial step to reduce the false positive rate of Type II lesions misclassified as Type I lesions (see also in result section 1). The macro-classifier CNN1 will then identify the individual image areas either positively or negatively for the Type I pathology features. Based upon the macro-classifier CNN1 prediction, the digital image areas are partitioned into two separate data sets, classified as ‘Type I’ or ‘not Type I’ lesion. Subsequently, both the positively or negatively identified image areas are further analyzed at a microscopic level based on cell types, by either the CNN2 or the CNN3 micro-classifiers. Both CNN2 and CNN3 require that the digital image sections are tiled into individual image patches that are 80 × 145 pixels in size. In case a Type I lesion is detected after CNN1, then CNN2 analyzes these image patches further and classifies each image patch as either Type I Rim, Type I Core, Healthy Tissue, Miscellaneous, or Empty. The remaining image patches (not identified by CNN1) are in parallel further classified with CNN3 as either Type II, Type III, Healthy, Miscellaneous, or Empty.

The output of the implementation of the three CNNs use ‘raw image patch counts’ (RIPC) per classification category, which are sum of the tiled image patches for each classification. RPIC are classified, classification locations stored, and subsequently visualized as colored overlays on the digital image. Every classification category is represented by a different color for easy visual, qualitative analysis and result verification (See Fig. 1). The classifications with unique colors for the seven pathology classifications are then overlaid and smoothed with a node labeling algorithm^74^, with each image patch classification being considered an individual node, onto the original H&E stained scanned digital image. The digital image of the lung lobe with colored overlay is then inspected and verified by the user at a second human intervention checkpoint, and at this point the user can make changes before the final classification calculations are performed (Fig. 2). Ultimately, the final output of LIRA using the two human intervention checkpoints includes the enumeration of the various lesion types, as well as the calculation of the area of lung involvement per classification category (presented as RIPC and % lung involvement per category). The LIRA software will provide a plain text, comma separated values (CSV) file, that consists of an automated enumeration of the number of RIPC for every classification category (7 in total). With the individual RIPC per classification category, the software then calculates the total lung area RIPC. Based on these calculations, the estimated % involvement is automatically calculated for each classification category by using the RIPC number for each lesion type divided by the total tissue RIPC x 100. Both the RIPC for each classification, as well as the % of lung area for each classification, is presented to the user in a CSV file for each image. These output CSV files can be opened on any personal computer and will be displayed as an Excel file. The LIRA source code with accompanying documentation is available from https://Github.com/TB-imaging/LIRA

### Neural network training

All CNNs in the LIRA Pipeline were trained using the Keras 2.0 machine learning framework in Python 3.5, on an NVIDIA Titan X GPU. Using a built-in human-in-the-loop classification tool for LIRA, trained research technicians assisted in creating the training set for CNN1 by collecting 791,000 individual image patches with a pixel size of 128 × 128. The purpose of these patches was solely to identify the Type I lesion structure at a macroscopic level. Additionally, 1.2 and 1.3 million image patches with a pixel size of 80 × 145 were collected in a similar manner for further classification at a microscopic level for both the Type 1 (CNN2) and Non-Type 1 lesion classification (CNN3), respectively. The image patches were generated as follows. In an initial step, the experienced technicians or pathologists performed manual labeling of the various classifications on an intact image. The specific regions of interests (ROI) for each classification type were subsequently extracted from the original image. The cropped ROIs were subsequently further divided into individual image patches. The reason for generating image patches for training from specific regions of interests (ROI) was primarily to prevent multiple conflicting labels being applied to a single lesion type. Due to the availability of data, not all lesion types were represented equally, which thereby created an unbalanced dataset. For training, we therefore did not use all generated image patches but these were random selected.

Each CNN model was subsequently trained on the provided training set of image patches, with the following parameters as implemented in Keras 2.0. Values for training and validation were initially chosen using a Bayesian Optimization Algorithm and values were further adjusted by hand. The composition of each of the neural networks is presented in the Supplemental Information, Tables 1 and 2.

### Validation of software

After training the CNNs, a validation step was included using a separate digital image set. Validation of the entire LIRA pipeline was performed by comparing results to a classical manual pathology readout. First, the performance of the LIRA software was assessed to recognize the various lesion types, i.e., calculation of the percent error per lesion type by LIRA when compared to the analysis by the experienced TB researcher (as base line). Second, the percent agreement between four board-certified veterinary pathologists (American College of Veterinary Pathologists) was calculated by comparing the results after classifying image patches with and without LIRA assistance. Lastly, the histopathology readout was measured more robustly by calculating a reliability coefficient developed to measure the agreement among observers, using the Krippendorff’s alpha coefficient^75^. The coefficient was calculated and based on data generated by four board-certified veterinary pathologists with and without the use of LIRA, as well as four research technicians with the use of LIRA.

To assess the agreement in the analysis by the pathologists with and without the use of LIRA, seven randomly selected scanned images of single lung lobes were selected for the software validation step. In a first step, the pathologists hand labeled each 80 × 145 pixel image patch using one out of seven classifications (same as described for LIRA). Data on every 80 × 145 pixel sized image patch classification was collected as RIPC. The hand labeled result by the pathologists and technicians generated a 7-color classification overlay for every digital image. The result also included the calculated RIPC for each of the seven classification categories for every digital image of a single lung lobe. In a second step, seven days later, the pathologists and research technicians were presented with the same seven digital images of lung lobes to review, this time with the assistance of LIRA predictions for both the macroscopic (CNN1) and microscopic classifications (CNN2, CNN3). Both the pathologists and technicians were requested to review the LIRA predictions, and to intervene and potentially modify the Type I lesion detection at the first intervention step (CNN1 LIRA prediction) and/or the individual image patch classifications at the second intervention step (CNN2, CNN3 LIRA prediction), if needed.

The analysis of the validation results included the calculation of the percent agreement in prediction results between the four pathologists^76^. Using percent agreement does not take into account that classifications may have been selected by chance, it is however still a commonly used metric and useful as a first measurement in the context of this work^77,78^.

As mentioned before, the classical histopathology readout is only semi-quantitative and, in some cases, subjective, which makes comparisons of the LIRA prediction results to a gold standard unreliable. To study the variance in readouts between the different pathologists after manually reviewing the digital images, we opted for an inter annotator comparison approach to calculate the agreement among the pathologists with and without LIRA. The Krippendorff’s alpha coefficient calculation using nominal data and any number of observers was implemented here as an analysis tool to quantify the agreement between the prediction results of multiple observers with or without assistance with LIRA. The Krippendorff’s alpha approach takes into account chance classifications, can be used with nominal data, using more than two raters, with multiple categories, plus the approach can be used with large datasets^79^. Each image patch was considered as a separate observation, and the classifications from all four pathologists were used for calculations. The Krippendorff’s alpha approach provides a coefficient between 0 and 1, with α < 0.667 being rejected, α ≥ 0.667 being the lowest acceptable limit, and α ≥ 0.800 being considered to have good agreement^80^.

### Evaluation metrics for performance and accuracy of the neural networks

For CNN1 the sensitivity was calculated by $\frac{True\,Positive}{(True\,Positive+False\,Negative)}\ast 100$ and specificity was calculated using$\,\frac{True\,Negative}{(True\,Negative+False\,Positive)}\ast 100$. These numbers were acquired by counting the number of true positives, true negatives, false positives, and false negatives within 70 digital images of lung lobes that had been classified by the CNN1 detector. This approach did not stipulate that the entire lesion was detected, but any area of the lesion be correctly or incorrectly identified.

In order to determine the accuracy of CNN2 and CNN3, a standard or ground truth classification was established on 12 digital images by a researcher with substantial experience analyzing C3HeB/FeJ mouse pathology (author B.C.A.). The researcher manually labeled each image patch using one of the seven different classifications described earlier. The researcher was also the main individual training LIRA, and therefore this analysis was mainly aimed to investigate whether LIRA performed adequately and met expectations. The 12 digital images containing a lung lobe consisted of three images primarily composed of Healthy Tissue, three images primarily composed of Type I lesions, three images composed of Type II images, and three images composed primarily of Type III lesions. Total image patch counts for only the classification of interest were then used to calculate the percent error for each individual classification for LIRA compared to the manual labeling result by the researcher.

## Supplementary information

Supplementary information.

## Data Availability

The LIRA computer code was written in Python programming language, and is provided open source to the research community without any restrictions from https://Github.com/TB-imaging/LIRA, including the full source code, documentations and usage guidelines.
