# Supplementary material for: Digital Image Analysis of Heterogeneous Tuberculosis Pulmonary Pathology in Non-Clinical Animal Models using Deep Convolutional Neural Networks
Source: Sci Rep. 2020 Apr 8;10:6047. doi: 10.1038/s41598-020-62960-6 (PMC7142129; doi:10.1038/s41598-020-62960-6)
Supplement: Supplementary file 1 — Supplementary information. [file 41598_2020_62960_MOESM1_ESM.pdf]

SCIENTIFIC REPORTS

SREP-19-31990A

Digital Image Analysis of Heterogeneous Tuberculosis Pulmonary Pathology in Non-Clinical Animal Models using Deep Convolutional Neural Networks

Authors:

Bryce C. Asay[1], Blake Blue Edwards[1,2], Jenna Andrews[1], Michelle E. Ramey[1], Jameson D. Richard[1], Brendan K. Podell[1], Juan F. Muñoz Gutiérrez[1], Chad B. Frank[1], Forgivemore Magunda,[1] Gregory T. Robertson[1], Michael Lyons[1], Asa Ben-Hur[2] and Anne J. Lenaerts[1*]

Affiliation:

1) Mycobacteria Research Laboratories, Department of Microbiology, Immunology and Pathology, Colorado State University, Fort Collins, Colorado, United States of America

2) Department of Computer Science, Colorado State University, Fort Collins, Colorado, United States of America

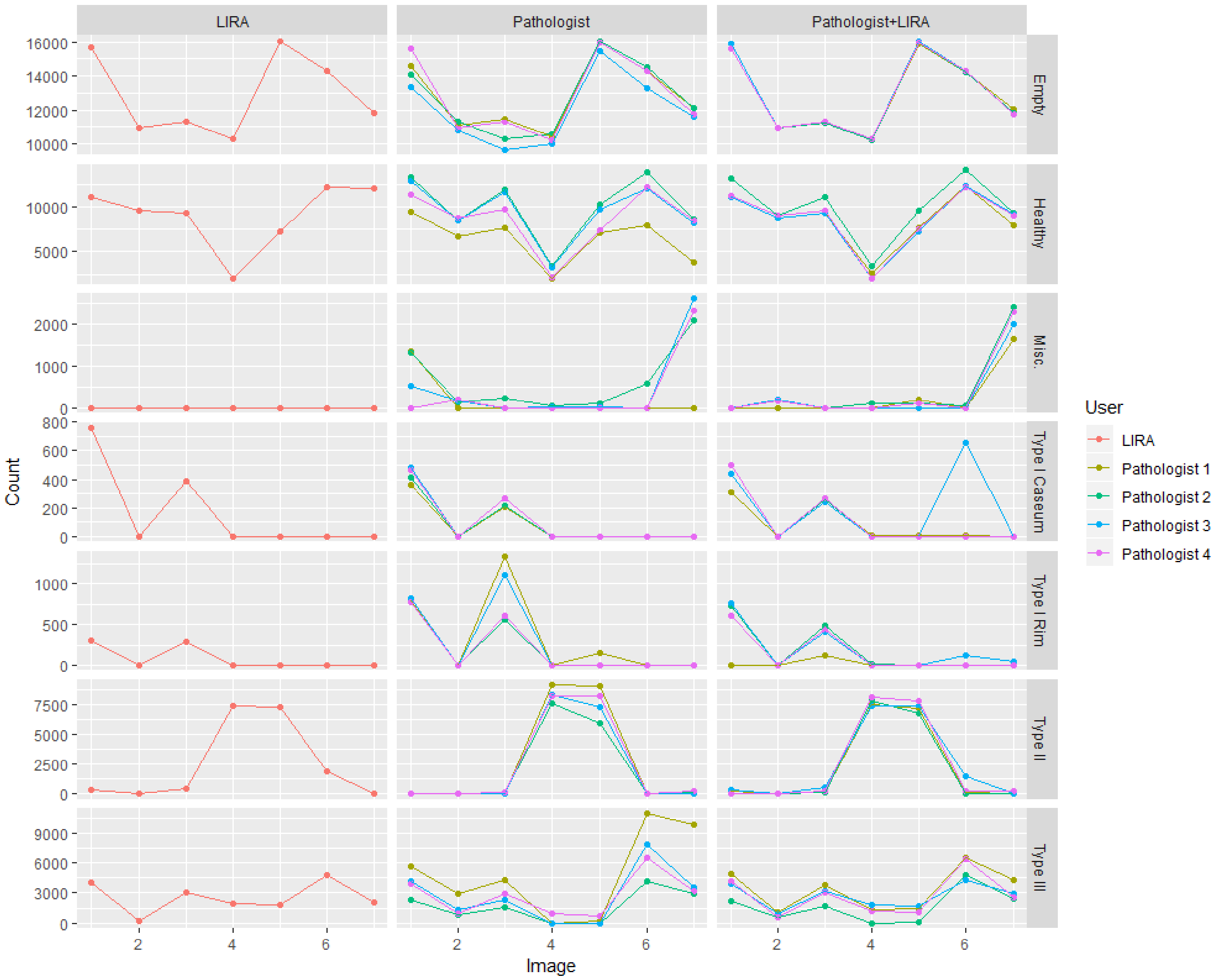

Supplemental Figure 1. Line graph representation of the pathology readouts for 7 digital images using 7 classifications: using LIRA, manual labeling by a pathologist, and the pathologist readout with LIRA assistance. Left Y axis depicts the Raw Image Counts generated during classifications, Right Y axis represents each of the pathology classifications, and the X axis shows the image number. Each colored line represents an individual pathologist or LIRA. As visualized, there is more agreement when the pathologists are assisted by LIRA (Pathologist + LIRA) than when the pathologists are not using LIRA (Pathologists). Readouts by Pathologists + LIRA are only marginally different than the classifications proposed by LIRA.

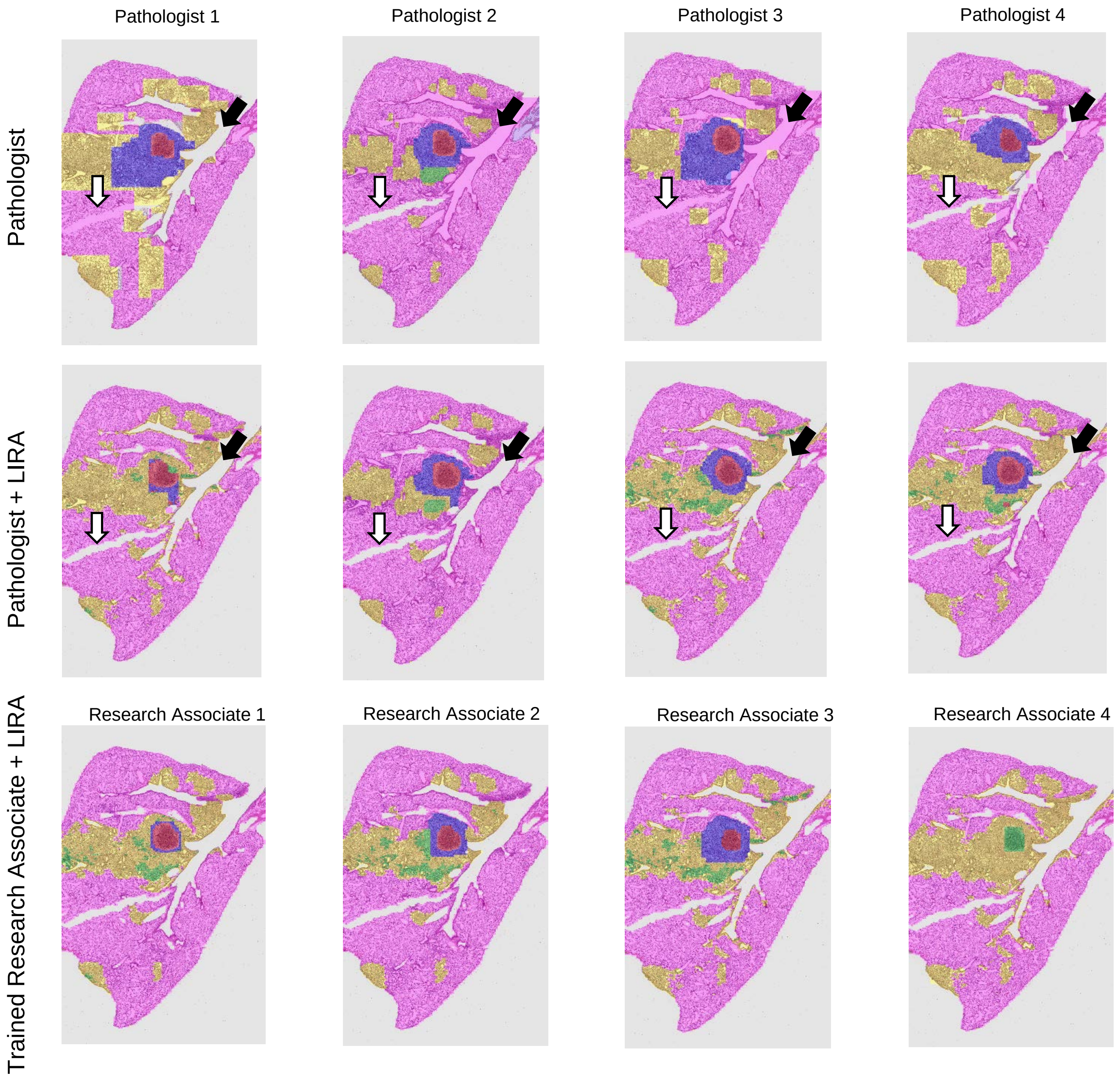

Supplemental Figure 2. Detailed Representation of the Pathology Analysis of a single image after hand-labeling by a pathologist with and without assistance of LIRA and by a trained research associate using LIRA. Analysis of Digital Image 3 which consists of a single Type I Core (Red), Type I Rim (Blue), Type II lesions (Green), multiple Type III (Yellow), and Healthy Tissue (Pink). The top row consists of pathologists hand labeling the tissue, the second row consists of pathologists with the assistance of LIRA, and the third row is the labeling by the research associates with the assistance of LIRA. Black arrows are marking bronchial airway spaces and white arrow shows processing artifacts.

Supplemental Table 1. CNN Parameters and Values

Classifier	Parameter	Value
CNN 1	Epoch	1000
	Loss-Function	Cross-Entropy
	Optimizer	Adam<br>Learning Rate= 10^{-4}
	Regularization	L2 : 10^{-4}.
	Mini-Batch Size	100
CNN 2&3	Epoch	10
	Loss-Function	Cross-Entropy
	Optimizer	Adam<br>Learning Rate= 10^{-4}
	Regularization	L2: 10^{-5}
	Mini-Batch Size	100

Supplemental Table 2. Description of the specifics on the creation Modular Network model.
The first table shows the specifics of the macroscopic Type 1 detector (CNN1).
The bottom table contains the information for the two microscopic CNNs (CNN2&3).

CNN 1

Layer Type	Filter Size	Stride	Activation Function	Input Size	Output Size
Convolution	7x7x16	1x1	ReLU	128x128x3	128x128x16
Max Pooling	2x2	2x2	N/A	128x128x16	64x64x16
Convolution	7x7x32	1x1	ReLU	64x64x16	64x64x32
Max Pooling	2x2	2x2	N/A	64x64x32	32x32x32
Convolution	3x3x32	1x1	ReLU	32x32x32	32x32x32
Convolution	3x3x64	1x1	ReLU	32x32x32	32x32x64
Max Pooling	2x2	2x2	NA	32x32x64	16x16x64
Convolution	3x3x64	1x1	ReLU	16x16x64	16x16x64
Convolution	3x3x128	1x1	ReLU	16x16x64	16x16x128
Max Pooling	2x2	2x2	N/A	16x16x128	8x8x128
Convolution	3x3x64	1x1	ReLU	8x8x128	8x8x64
Average Pooling	8x8	8x8	N/A	8x8x64	1x1x64
Flatten	N/A	N/A	N/A	1x1x64	64
Dense/Output	N/A	N/A	Softmax	64	2

CNN 2&3

Layer Type	Filter Size	Stride	Activation Function	Input Size	Output Size
Convolution	7x12x20	1x1	Sigmoid	80x145x3	74x134x20
Max Pooling	2x2	2x2	N/A	74x134x20	37x67x20
Convolution	6x10x40	1x1	Sigmoid	37x67x20	32x58x40
Max Pooling	2x2	2x2	N/A	32x58x40	16x29x40
Flatten	N/A	N/A	N/A	16x29x40	18560
Fully Connected	N/A	N/A	Sigmoid	18560	1024
Fully Connected	N/A	N/A	Sigmoid	1024	100
Fully Connected	N/A	N/A	Softmax	100	4
